# Editorial: Micro-nano-materials for drug delivery, disease diagnosis, and therapeutic treatment

**DOI:** 10.3389/fbioe.2025.1645112

**Published:** 2025-09-01

**Authors:** Bing Guo, Tingting Peng, Chao Mi, Lei Ye

**Affiliations:** ^1^ School of Science, Harbin Institute of Technology, Shenzhen, China; ^2^ State Key Laboratory of Bioactive Molecules and Druggability Assessment, Jinan University, Guangzhou, China; ^3^ School of Advanced Engineering, Great Bay Institute for Advanced Study, Great Bay University, Dongguan, Guangdong, China; ^4^ Department of Biomedical Engineering, University of Alabama at Birmingham, Birmingham, AL, United States

**Keywords:** micro-nano-materials, drug delivery, diagnosis, theranostics, therapeutics

Micro- and nano-sized platforms have shown high efficiency in the delivery of diagnostic and therapeutic drugs for the treatment of different diseases such as tumors, inflammation, fibrosis, injury, infections, senility, neurological disorders, etc. (Liu et al., 2025; Huang et al., 2024) The merits of micro- and nano-sized platforms, including ease in composition and surface modification, controlled drug release manner, and active/passive targeting effects, synergistically contribute to the decreased threshold therapeutic dosage, minimized side effects, and ultimately improved prognosis for patients (Roy et al., 2023a; Roy et al., 2025). It is a great honor that Frontiers in Bioengineering and Biotechnology we launched this Research Topic, “Micro-Nano-Materials for Drug Delivery, Disease Diagnosis, and Therapeutic Treatment,” which would include the recent advances in micro-nano-materials with a special application in drug delivery and disease diagnostics and therapeutics. These emerging research topics have attracted increasing attention among scientists. Importantly, the recently developed micro-nano-materials have shown great promise to translate from bench to bed side, because they could be customized to bypass the physiological and pathological barriers, increase targeting efficiency, minimize off-target effects, optimize pharmacokinetics (PK) and pharmacodynamics (PD), decrease the therapeutic threshold dosage and side-effects, and ultimately boost the treatment outcomes and prognosis (Zhang et al., 2022; Chen et al., 2025). In this Research Topic, both reviews and original research articles are collected with the focused Research Topic.

So far, cancer is still one of the most life-threatening diseases for human beings worldwide. Although a great number of treatment paradigms and drugs have been invented, patients bearing cancer often suffer from severe side effects and a poor prognosis. For efficiently diagnosing cancer, the fluorescence imaging in the second near-infrared window (NIR-II) offers advantages such as deep tissue penetration, reduced autofluorescence, high spatiotemporal resolution, and excellent signal-to-background ratio, making it a powerful tool for diagnosis and imaging-guided drug delivery (IGDD) in cancer therapy. Roy et al. highlighted recent advancements in NIR-II IGDD systems, focusing on strategies to enhance drug loading, controlled release, and overcoming biological barriers. It categorizes these systems based on design elements, including intrinsic or extrinsic fluorophores and responsive drug delivery mechanisms. Moreover, they discussed the applications of NIR-II IGDD in various treatment modalities, such as chemotherapy, photodynamic, photothermal, chemodynamic, immunotherapy, gas therapy, and ion channel-targeted therapy, while also emphasizing multifunctional capabilities like real-time monitoring of drug release and therapeutic effects (Roy et al., 2023b). Isaí et al. used Coffea arabica green bean extract, doxorubicin, and commercial organic ligands (MUA) to synthesize gold nanoparticles through a green synthetic route. The nanoparticles showed good stability with a diameter of 40 nm at a physiological pH environment, but triggered release at tumoral pH microenvironment, and exhibited highly efficient tumor specificity and cytotoxicity to H69 lung cancer cells. This novel approach provides a promising avenue for cancer therapy by harnessing the distinct advantages of each component to enhance treatment effectiveness while minimizing adverse side effects Trejo-Teniente et al.

Nowadays, with the increasing global aging population, osteoarthritis (OA) with closely related to aging and obesity, has become a representative degenerative disease and leads to an economic burden to society. The research in OA treatment based on nanomedicines has grown rapidly. The research direction generally is to customize nanocarriers to efficiently deliver therapeutic agents like drugs and nucleic acids to the pathological lesion of OA, in which the microenvironment is weakly acidic and full of synovial fluid. For example, hybrid DDS platforms combining hydrogels, liposomes, and particle-based carriers are being designed to localize within the synovial joint and alleviate pain and inflammation. Meanwhile, cartilage-targeting systems are being engineered to bind specific intra-cartilage components such as aggrecans, collagen type II, and chondrocytes, improving drug access to cellular and intracellular targets. These strategies aim to enable effective delivery of disease-modifying OA drugs, including emerging gene therapies. Recent clinical progress includes the approval of steroid-loaded polymeric microparticles for prolonged pain management. In preclinical studies, electrically charged biomaterials have demonstrated improved cartilage targeting and therapeutic outcomes. Ongoing clinical trials assessing viral vector-based gene therapies hold promise for durable, long-term treatment solutions for OA (Mehta et al., 2021). Shuai et al. updated the recent progress of pH-responsive nanomedicines for OA therapy, including the composition, synthetic routes, pH-responsive mechanism, targeting capability, and application examples. Special focus is on how to achieve targeting to the acidic microenvironment of the inflamed joints of OA. This work also emphasizes the integration of multiple stimuli-responsive mechanisms, such as pH, ultrasound, magnetic fields, and MMPs, to overcome the limitations of burst release and poor targeting seen in conventional DDS. A significant innovation lies in combining therapy and imaging functionalities, enabling real-time monitoring of disease progression. The paper also highlights underexplored targets like synovium and subchondral bone, encouraging a shift beyond cartilage-centric strategies. Moreover, it introduces advanced dual-drug delivery platforms capable of co-loading hydrophilic and hydrophobic drugs, achieving synergistic anti-inflammatory, antioxidant, and cartilage-repair effects. The inclusion of multifunctional materials like circular brush zwitterionic polymers with joint lubrication and ROS-scavenging properties further enhances therapeutic performance. Finally, the work underlines the need to tailor nanoparticle design to OA’s complex microenvironment, promoting the development of highly specific, biocompatible, and efficient nanocarriers for precise and multimodal OA therapy Liao et al.

Recently, ionic liquid (IL)-based carriers as a new drug delivery platform have gained increasing attention in the pharmaceutical industry, owing to their merits including versatile chemical structures, ease in synthesis, good stability, and amphiphilic nature with efficient solubility to both hydrophilic and hydrophobic drugs. For example, the ILs like organic salts with melting points below 100 °C can serve as solvents, excipients, or active pharmaceutical ingredients (API-ILs), with some API-ILs combining drug molecules with biologically meaningful ions to improve pharmacokinetics and minimize polymorphism Research Topic (Berton and Shamshina, 2023). Ankit et al. offered a timely summary of IL-based carriers for drug delivery applications. The authors pointed out that these carriers could offer colloidal stability with drug encapsulation and, more importantly, assist in breaching physiological and pathological barriers, and enrich drug concentration in lesion sites, ultimately improving the treatment outcomes. In addition, the authors showcase the challenges for IL-based carriers such as impurity, toxicity, degradation, stability, and even skin permeability, and offer possible solutions to overcome the shortcomings. This work highlights the novel integration of structurally tunable ILs with nanotechnology to create multifunctional, biocompatible drug delivery systems that enhance therapeutic efficacy, permeability, and precision while addressing critical safety and regulatory challenges for clinical translation Jain et al.

At present, thalassemias and other hematological abnormalities and diseases are still clinical Research Topic with insufficient treatment paradigms. Importantly, gene therapy is one of the most efficient therapeutic approaches, and the hematopoietic stem and progenitor cells (HSPCs) are the desirable targeting sites. However, due to a lack of efficient delivery vectors, gene therapy often suffers from off-target effects. Samik et al. herein reported an innovative delivery system based on engineered human megakaryocytic extracellular vesicles (huMkEVs), and synthetic hybrid semi-synthetic vesicles based on liposome and huMkEVs. The synthesized vectors showed prolonged circulation, efficient targeting to lesion sites, strong tropism of huMkEVs for murine HSPCs, and excellent treatment efficacy on hematological diseases. This work uniquely demonstrates that huMkEVs, both native and engineered, can specifically target HSPCs to promote megakaryopoiesis and serve as versatile, modifiable delivery vehicles for functional cargo, offering a promising new therapeutic strategy for treating thrombocytopenia and other hematological disorders Das et al.

As editors, we hope this Research Topic will pique interest among a wide audience and serve as a spark to novel ideas and efforts for researchers with different backgrounds.
